# A Change

**Published:** 1883-07

**Authors:** 


### A CHANGE.

Dr. Roswell Park, of Chicago, has assumed the editorial management of *The Weekly Medical Review.* A sterling journal will thus have a capable man at the helm, and we desire to extend congratulations and best wishes to both the high contracting parties.

